# Antioxidant Vitamins and Carotenoids Intake and the Association With Poor Semen Quality: A Cross-Sectional Analysis of Men Referring to an Italian Fertility Clinic

**DOI:** 10.3389/fnut.2021.737077

**Published:** 2021-10-04

**Authors:** Valentina De Cosmi, Fabio Parazzini, Carlo Agostoni, Stefania Noli, Sonia Cipriani, Irene La Vecchia, Stefania Ferrari, Giovanna Esposito, Francesca Bravi, Elena Ricci

**Affiliations:** ^1^Department of Clinical Sciences and Community Health, University of Milan, Milan, Italy; ^2^SIGENP (Italian Society of Pediatric Gastroenterology, Hepatology, and Nutrition), Milan, Italy; ^3^Department of Woman, Newborn and Child, Fondazione IRCCS Ca' Granda Ospedale Maggiore Policlinico, Milan, Italy; ^4^Pediatric Intermediate Care Unit, Fondazione IRCCS Ca' Granda Ospedale Maggiore Policlinico, Milan, Italy

**Keywords:** antioxidants, assisted reproduction techniques, carotenoids, diet, micronutrients

## Abstract

Several studies suggested that male's diet affects fertility. This cross-sectional analysis from a prospective cohort study aims to explore the relation between antioxidants intake and sperm parameters in sub-fertile couples referring to a Fertility center. Socio-demographic characteristics, health history, lifestyle habits, and diet information were obtained. A semen sample was analyzed to proceed with assisted reproduction. Three hundred and twenty-three men were enrolled: 19.1% had semen volume (SV) < 1.5 mL, 31.4% sperm concentration (SC) < 15.0 mil/mL, 26.8% sperm motility < 32%, and 33.0% had total sperm count (TSC) < 39.0 mil. Higher levels of α-carotene were associated to lower risk of low SC [4th vs. 1st quartile, adjusted OR (aOR) 0.43, 95% CI 0.20–0.91) and low TSC (aOR 0.46, 95% CI 0.22–0.95). Higher intake of β-carotene was inversely associated with risk of low TSC. Lycopene intake was associated with higher risk for these conditions (aOR 2.46, 95%CI 1.01–5.98, SC), and (aOR 3.11, 95%CI 1.29–7.50, TSC). Risk of low semen volume was lower in men with higher level of vitamin D intake (aOR 0.25, 95%CI 0.09–0.66)]. Further research, especially, well-designed randomized clinical trials (RCT), is needed to understand how diet modifications may have a role in modulating male fertility and fecundability.

## Introduction

Approximately 15% of couples are affected by fertility problems, and male causes are responsible for about 30% of them (1, 2). Recently, a meta-analysis has shown that in European men sperm concentration has declined by an overall 32% over the past 50 years (3). In the majority of cases, sub optimal semen quality is idiopathic, with no evident explanation for compromised spermatogenesis. Even if growing evidence suggests that environmental conditions and lifestyle habits may affect semen quality (4), the causal link between impaired male fertility and environmental factors is still uncertain. Besides genetic and endocrine factors (5, 6), an important contribution derives from lifestyle (such as smoking, overweight, physical activity, alcohol intake, diet) (7–9).

A comprehensive review concluded that healthy diets, including nutrients such as some antioxidants (vitamin E, vitamin C, β-carotene, selenium, zinc, cryptoxanthin, and lycopene), vitamins (vitamin D and folate), and omega-3 fatty acids, but with low saturated fatty acids and trans-fatty acids intake, has been positively associated to good sperm quality parameters (10). On the contrary, a Cochrane meta-analysis (11) found that randomized controlled trials of treatments with antioxidants vs. placebo did not indicate an influence on sperm parameters. It is possible that a diet rich in antioxidants is related with healthy lifestyle, with a consequent positive effect on semen quality. Thus, to analyze the association between diet and sperm quality is of specific interest also to better understand the role of antioxidants on sperm parameters. Available data on this potential association is limited (10–12).

Despite this, numbers of dietary supplements (DS) have been suggested to ameliorate sperm parameters and male fertility. Most contain a large number of ingredients, often supported by poor scientific evidence or below their minimal effective daily dose (13). Zinc is the ingredient commonly found, followed by selenium, arginine, coenzyme Q, and folic acid. Garolla et al. examined the composition of 21 DS in the Italian market (14). Authors showed that in each supplement the mean number of ingredients was higher than 7 (from 2 to 17) and that 13 DS contained at least one ingredient without any proof of efficacy (i.e., astragalus, taurine, and riboflavin) (14). To offer additional evidence on the relation between antioxidant vitamins and carotenoids intake and the risk of poor semen quality, we analyzed data from a study on the relationship between lifestyle patterns and sperm parameters in men of sub-fertile couples, presenting to an Italian Fertility Clinic and candidate to assisted reproductive procedures. A novelty of this study is the fact that it includes a Southern European population, usually characterized by frequent intake of fruit and vegetables.

## Materials and Methods

From September 2014 to December 2016, subfertile couples, entered for evaluation to the Fertility Unit of Fondazione IRCCS Ca' Granda, Ospedale Maggiore, Policlinico, Milan, and eligible for assisted reproduction technologies (ART), were asked to participate into an ongoing prospective cohort study on the role of lifestyle habits and diet on ART outcome. The study protocol was approved (reference number 2616, December 9, 2014) by the Ethical Review Board of Fondazione IRCCS Ca' Granda, Ospedale Maggiore, Policlinico (Milan). All procedures were in accord with the Helsinki Declaration and all participants gave written informed consent. The study was explained over the diagnostic phase. On the oocyte retrieval day, centrally trained personnel interviewed both partners using a standard questionnaire (Supplementary Material) to collect information on general socio-demographic characteristics, health history, and lifestyle habits (including smoking, physical activity, alcohol intake, and caffeine consumption).

On the same day, a semen sample was also collected and analyzed to proceed with *in vitro* fertilization (IVF) or intra-cytoplasmic sperm injection (ICSI). In the present study, only evidence from the male partner was reported. Couples that could not speak Italian were excluded from the study. The participation rate was close to 95%. This high participation rate was principally because couples were interviewed during the period spent waiting for the different diagnostic stages before the actual ART procedure. Considering both this down time and the not sensitive character of questions, couples did not usually refuse to answer the questionnaire.

History of previous chemo- or radiotherapy, and of previous reproductive organ diseases (ROD), like orchiectomy, cryptorchidism, and varicocele was retrieved from medical reports. Men with one of the above-mentioned conditions were classified as having risk factors for impaired fertility.

Through the World Health Organization (WHO)'s indications we classified body mass index (BMI) (15). We categorized occupational physical activity (PA) as heavy (or very heavy), light/moderate, mainly standing or mainly sitting. We described leisure PA in term of hours/week: <2, between 2 and 4, ≥5, but no details on intensity or type of leisure PA were collected. As regards smoking habits, we created the following categories: never, former, or current, and we registered the number of cigarettes smoked daily, and duration of smoking. Information on alcohol intake was collected as usual weekly consumption (1 unit = 125 mL wine or 330 mL beer or 30 mL spirits, all containing ~12.5 g of ethanol). An intake lower than one unit per week was coded as 0.5. Caffeine intake from coffee (60 mg per cup), cappuccino (75 mg per cup), tea (45 mg per cup), decaffeinated coffee (4 mg per cup), and chocolate (6 mg/10 g) was calculated (16).

A previously validated food frequency questionnaire (FFQ) (17–19) was used to obtain information on diet. Patients' usual weekly food consumption in the last year was asked. The FFQ includes the average weekly consumption of 78 food items or food groups (including the major sources of animal fats—i.e., red meat, milk, cheese, ham, salami—folates, vitamins—vegetables and fruit—pasta and bread consumption, cake, sweets and chocolate, fish) and beverages. Intakes lower than once per week, but at least once per month, were coded 0.5 per week. To account for seasonal consumptions we considered weekly consumption of vegetables/fruits available in limited periods during the year, weighted for months of consumption. Daily energy and mineral, macro- and micronutrient intake was estimated using the most recent update of an Italian food consumption database (20).

### Sperm Analysis

Two to five days of abstinence was requested to men before semen analysis. Samples were obtained by masturbation and collected into a sterile plastic container provided and labeled with the date and time of collection. Then, they were conserved at room temperature until complete liquefaction. All the seminal fluid examinations were carried out by the laboratory of the Unit. Duration of complete liquefaction (<1 h) was registered, until 1 h was reached. Semen analysis was performed using Makler counting chamber (concentration and motility) and by following standardized methods according to the WHO guidelines (2010). Sperm count was defined after a minimum of two repeated measurements. All biologists have been certified by the European Society of Human Reproduction and Embryology (ESHRE) on semen analysis. Our laboratory participates to external quality assessment (VEQ) managed by Azienda Ospedaliera Universitaria Careggi (Firenze, Italy). Volume (mL), sperm concentration (spermatozoa N/mL), and motility (%) were taken into consideration. Sperm motility was classified into total (progressive + non-progressive motility) and progressive motility. Total sperm count was calculated as volume × sperm concentration. The WHO semen analysis manual were used to compare sperm volume, concentration, total count, and motility (15) with the given reference values: 1.5 mL for volume, 15.0 millions/mL for concentration, 39.0 million for total count and 32% for sperm motility. Men who had at least one recorded parameter were included in the analysis. According to the center procedure, if sperm concentration was lower than 1 million/mL motility was not evaluated, as the couple was candidate to ICSI, and motility value was missing for the subject.

As semen samples were collected specifically to carry out ART procedures, sperm morphology was only evaluated in partners of those couples undergoing IVF and after semen capacitation (and not on fresh sperm samples). All semen samples underwent Density gradient centrifuge (DGC) method; the swim-up procedure is subsequently applied depending on sperm concentration. The laboratory personnel were trained using the European Society of Human Reproduction and Embryology (ESHRE) Special Interest Group in Andrology Basic Semen Analysis Course (21).

### Statistical Analysis

Categorical or ordinal variables were described as frequency (%), continuous variables as means (standard deviation, SD) if normally distributed and medians (interquartile range, IQR) if not. Comparisons were performed using chi-square or Mantel-Haenszel test, as appropriate, for categorical variables, Student's *t*-test or analysis of variance if more than two classes were present, for normally distributed continuous variables, and Mann-Whitney *U*-test for not normally distributed continuous variables. Four domains of semen quality were assessed: volume, concentration, total count, and motility.

We estimated the odds ratios (ORs) and the corresponding 95% confidence intervals (CIs) for semen volume < 1.5 mL, sperm concentration < 15.0 mil/mL, sperm motility < 32%, and total sperm count < 39.0 mil, in quartiles of antioxidants intake, and the corresponding trend. To account for potential confounders, we included terms for variables associated to each micronutrient or sperm parameter in the unconditional logistic regression models. Moreover, factors previously associated with sperm quality were included in the equation (age, alcohol intake, current smoking, days of abstinence). Factors used for adjustment are indicated in table footnotes. All the analyses were performed with the SAS software, version 9.4 (SAS Institute, Inc., Cary, NC, USA).

## Results

**Figure 1:**
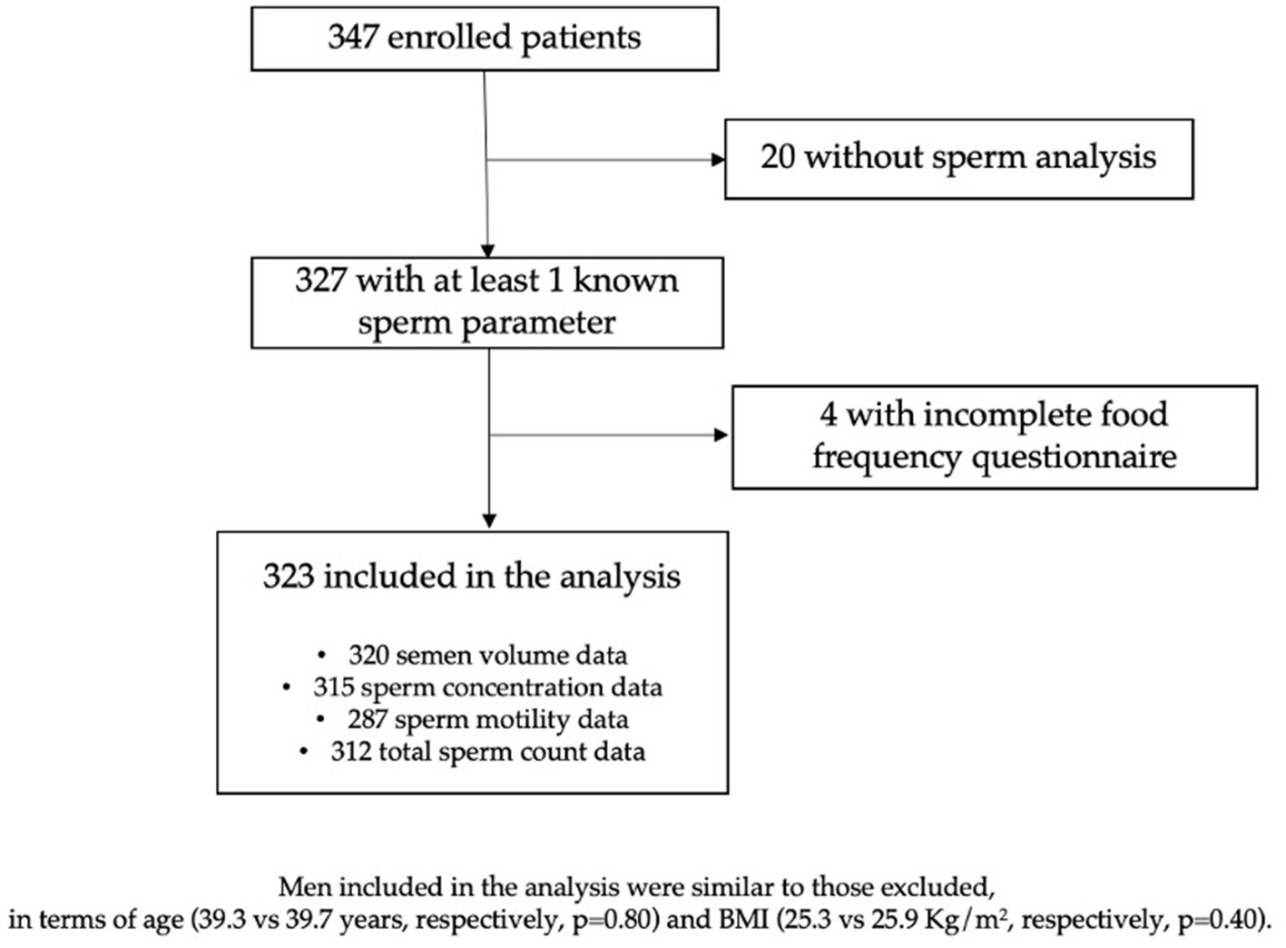
Flow chart of enrolled patients and reasons for exclusion.

Three hundred forty-seven men were interviewed: 327 (94.2%) had at least one seminal parameter measured and, among them, 4 did not provide complete information about lifestyle and were excluded from the analysis (Figure 1). Therefore, the concluding analyses were led on 323 men, aged 39.3 years on average (SD 5.3, range 27–60): 61/320 (19.1%) had low semen volume, 99/315 (31.4%) had low concentration, 77/287 (26.8%) had low sperm motility, and 103/312 (33.0%) had low total sperm count. Prevalence was 46% for overweight and 8.7% for obesity (BMI ≥ 30.0); 31.7% of men were current and 28.9% former smokers. Table 1 depicts antioxidants and micronutrients intake of men, according to presence of sperm abnormalities. As presented, at this univariate analysis, we found that low semen volume was associated with Vitamin D intake (*p* = 0.002), low sperm concentration with α-carotene and lycopene (*p* = 0.05 and 0.007, respectively), low sperm motility with β-cryptoxanthins (*p* = 0.04), and lutein (*p* = 0.03), low total sperm count with α- and β-carotene (*p* = 0.017 and 0.056, respectively), and with lycopene intake (*p* = 0.013). Table 2 shows the demographic characteristics and lifestyle habits of men, according to sperm characteristics. At this cross-sectional univariate analysis, low semen volume was frequent in older age classes (*p* = 0.016), low sperm concentration and total count in men with a history of ROD (*p* = 0.0001 and *p* = 0.0002, respectively), low sperm motility in men with history of ROD (*p* = 0.01), and with low level of occupational PA (*p* = 0.048). Days of abstinence were significantly higher in men with low motility (*p* = 0.026).

**Table 1:** Antioxidants and micronutrients intake of 323 men undergoing assisted reproduction technique, according to presence of sperm abnormalities.

**Micronutrients: quartiles**	**Semen volume** ***N*** **= 320**					**Sperm concentration** ***N*** **= 315**					**Sperm motility** ***N*** **= 287**					**Total sperm count** ***N*** **= 312**				
	**<1.5 mL** ***N*** **= 61 (19.1%)**		**≥1.5 mL** ***N*** **= 259 (80.9%)**		***p***	**<15.0 mil/mL** ***N*** **= 99 (31.4%)**		**≥15.0 mil/mL** ***N*** **= 216 (68.6%)**		***p***	** <32.0%** ***N*** **= 77 (26.8%)**		**≥32.0%** ***N*** **= 210 (73.2%)**		***p*** ^*^	** <39.0 mil** ***N*** **=** **103 (33.0%)**		**≥39.0 mil** ***N*** **= 209 (67.0%)**		***p***
	***N***	**%**	***N***	**%**		***N***	**%**	***N***	**%**		***N***	**%**	***N***	**%**		***N***	**%**	***N***	**%**	
Vitamin C																				
1st	19	31.1	62	23.9		22	22.2	55	25.5		21	27.3	51	24.3		28	27.2	49	23.4	
2nd	13	21.3	65	25.1		29	29.3	50	23.1		19	24.7	50	23.8		25	24.3	52	24.9	
3rd	17	27.9	64	24.7		25	25.3	55	25.5		16	20.8	57	27.1		29	28.2	51	24.4	
4th	12	19.7	68	26.3	0.28	23	23.2	56	25.9	0.86	21	27.3	52	24.8	0.77	21	20.4	57	27.3	0.31
Vitamin D																				
1st	25	41.0	55	21.2		23	23.2	53	24.5		22	28.6	47	22.4		29	28.2	47	22.5	
2nd	16	26.2	64	24.7		27	27.3	54	25.0		18	23.4	54	25.7		24	23.3	55	26.3	
3rd	9	14.8	70	27.0		24	24.2	55	25.5		20	26.0	53	25.2		26	25.2	52	24.9	
4th	11	18.0	70	27.0	**0.002**	25	25.3	54	25.0	0.96	17	22.1	56	26.7	0.32	24	23.3	55	26.3	0.40
Vitamin E																				
1st	13	21.3	67	25.9		23	23.2	54	25.0		23	29.9	51	24.3		25	24.3	51	24.4	
2nd	14	23.0	66	25.5		26	26.3	54	25.0		19	24.7	47	22.4		30	29.1	50	23.9	
3rd	15	24.6	64	24.7		25	25.3	52	24.1		18	23.4	54	25.7		22	21.4	54	25.8	
4th	19	31.1	62	23.9	0.23	25	25.3	56	25.9	0.91	17	22.1	58	27.6	0.21	26	25.2	54	25.8	0.68
α-carotene																				
1st	17	27.9	63	24.3		33	33.3	46	21.3		23	29.9	50	23.8		35	34.0	43	20.6	
2nd	15	24.6	65	25.1		22	22.2	54	25.0		20	26.0	50	23.8		25	24.3	51	24.4	
3rd	12	19.7	68	26.3		23	23.2	59	27.3		18	23.4	54	25.7		21	20.4	59	28.2	
4th	17	27.9	63	24.3	0.85	21	21.2	57	26.4	**0.05**	16	20.8	56	26.7	0.18	22	21.4	56	26.8	**0.017**
β-carotene																				
1st	17	27.9	63	24.3		28	28.3	51	23.6		24	31.2	49	23.3		33	32.0	45	21.5	
2nd	12	19.7	68	26.3		24	24.2	54	25.0		18	23.4	50	23.8		24	23.3	54	25.8	
3rd	16	26.2	64	24.7		27	27.3	52	24.1		21	27.3	53	25.2		25	24.3	52	24.9	
4th	16	26.2	64	24.7	0.95	20	20.2	59	27.3	0.25	14	18.2	58	27.6	0.10	21	20.4	58	27.8	**0.056**
Lycopene																				
1st	15	24.6	65	25.1		20	20.2	57	26.4		21	27.3	53	25.2		23	22.3	54	25.8	
2nd	19	31.1	61	23.6		19	19.2	62	28.7		24	31.2	50	23.8		18	17.5	62	29.7	
3rd	15	24.6	66	25.5		25	25.3	52	24.1		17	22.1	52	24.8		26	25.2	50	23.9	
4th	12	19.7	67	25.9	0.42	35	35.4	45	20.8	**0.007**	15	19.5	55	26.2	0.22	36	35.0	43	20.6	**0.013**
β-cryptoxanthins																				
1st	18	29.5	62	23.9		27	27.3	51	23.6		22	28.6	48	22.9		29	28.2	48	23.0	
2nd	16	26.2	64	24.7		16	16.2	61	28.2		17	22.1	55	26.2		20	19.4	56	26.8	
3rd	13	21.3	67	25.9		26	26.3	54	25.0		12	15.6	61	29.0		26	25.2	54	25.8	
4th	14	23.0	66	25.5	0.34	30	30.3	50	23.1	0.12	26	33.8	46	21.9	0.76^**^	28	27.2	51	24.4	0.99
Lutein																				
1st	18	29.5	62	23.9		27	27.3	50	23.1		27	35.1	42	20.0		31	30.1	46	22.0	
2nd	9	14.8	72	27.8		23	23.2	56	25.9		20	26.0	55	26.2		23	22.3	55	26.3	
3rd	16	26.2	63	24.3		24	24.2	55	25.5		11	14.3	58	27.6		23	22.3	55	26.3	
4th	18	29.5	62	23.9	0.64	25	25.3	55	25.5	0.67	19	24.7	55	26.2	**0.03**	26	25.2	53	25.4	0.36
Folates																				
1st	14	23.0	66	25.5		26	26.3	52	24.1		25	32.5	45	21.4		29	28.2	49	23.4	
2nd	16	26.2	65	25.1		20	20.2	57	26.4		16	20.8	60	28.6		22	21.4	55	26.3	
3rd	14	23.0	65	25.1		27	27.3	51	23.6		16	20.8	52	24.8		27	26.2	50	23.9	
4th	17	27.9	63	24.3	0.64	26	26.3	56	25.9	0.88	20	26	53	25.2	0.36	25	24.3	55	26.3	0.63

* *Mantel-Hanszel chi-square;*

** *heterogeneity chi-square p = 0.04. The bold values indicates p < 0.05*.

**Table 2:** Demographic characteristics and lifestyle patterns of 323 men undergoing assisted reproduction technique, according to presence of sperm abnormalities.

	**Semen volume** ***N*** **= 320**					**Sperm concentration** ***N*** **= 315**					**Sperm motility** ***N*** **= 287**					**Total sperm count** ***N*** **= 312**				
	**<1.5 mL** ***N*** **= 61 (19.1%)**		**≥1.5 mL** ***N*** **= 259 (80.9%)**		***p***	**<15.0 mil/mL** ***N*** **= 99 (31.4%)**		**≥15.0 mil/mL** ***N*** **= 216 (68.6%)**		***p***	**<32.0%** ***N*** **= 77 (26.8%)**		**≥32.0%** ***N*** **= 210 (73.2%)**		***p*** ^*^	**<** **39.0 mil** ***N*** **= 103 (33.0%)**		**≥39.0 mil** ***N*** **= 209 (67.0%)**		***p***
	***N***	**%**	***N***	**%**		***N***	**%**	***N***	**%**		***N***	**%**	***N***	**%**		***N***	**%**	***N***	**%**	
Age (years)																				
<35	8	13.1	51	19.7		14	14.1	45	20.8		15	19.5	40	19.0		15	14.6	43	20.6	
35–39	18	29.5	108	41.7		46	46.5	77	35.6		32	41.6	83	39.5		42	40.8	81	38.8	
≥40	35	57.4	100	38.6	**0.016**	49	39.4	94	43.5	0.14	30	39.0	87	41.4	0.77	46	44.7	85	40.7	0.43
Mean ± SD	41.2 ± 6.0		38.9 ± 4.9		**0.007**	39.1 ± 4.5		39.4 ± 5.5		0.65	39.4 ± 5.6		39.2 ± 5.1		0.70	39.6 ± 5.0		39.2 ± 5.3		0.49
College degree	28	45.9	102	39.4	0.35	38	38.4	89	41.2	0.64	34	44.2	84	40.0	0.53	43	41.8	83	39.7	0.73
ROD	10	16.4	54	20.8	0.43	32	32.3	30	13.9	**0.0001**	22	28.6	33	15.7	**0.01**	32	31.1	28	13.4	**0.0002**
BMI																				
<25.0	22	36.1	123	47.5		41	41.4	102	47.4		33	42.9	98	46.7		44	42.7	98	47.1	
25.0–29.9	34	55.7	113	43.6		48	48.5	96	44.6		37	48.1	95	45.2		50	48.5	93	44.7	
≥30.0	4	6.6	23	8.9	0.30	10	10.1	17	7.9	0.57	6	7.8	17	8.1	0.70	9	8.7	17	8.2	0.76
Smoking																				
Never	22	36.1	104	40.2		38	38.4	87	40.5		36	46.8	79	37.6		39	37.9	85	40.9	
Current	20	32.8	82	31.7		31	31.3	66	30.7		22	28.6	68	32.4		30	29.1	67	32.2	
Former	19	31.1	72	27.8	0.81	30	30.3	62	28.8	0.94	19	24.7	62	29.5	0.39	34	33.0	56	26.9	0.54
Occupational PA																				
Heavy	18	29.5	48	18.5		26	26.3	41	19.1		10	13.0	47	22.4		27	26.2	39	18.8	
Moderate	11	18.0	55	21.2		19	19.2	45	20.9		14	18.2	46	21.9		18	17.5	44	21.1	
Mainly standing	8	13.1	39	15.1		9	9.1	36	16.7		14	18.2	31	14.8		12	11.6	33	15.9	
Mainly sitting	23	37.7	117	45.2	0.08	45	45.4	93	43.3	0.20	38	49.4	86	41.0	**0.048**	46	44.7	92	44.2	0.37
Leisure PA																				
<2 h/week	27	44.3	105	40.5		39	39.8	92	43.2		27	35.1	89	42.4		48	47.5	82	39.6	
2–4 h/week	21	34.4	89	34.4		34	34.7	74	34.7		29	37.7	71	33.8		34	33.7	72	34.8	
≥5 h/week	11	18.0	63	24.3	0.32	25	25.5	47	22.1	0.77	18	23.4	49	23.3	0.62	19	18.8	53	25.6	0.13
Abstinence (days), median (IQR)	3	3–4	3	3–4	0.13	4	3–4	3	3–4	0.24	4	3–5	3	3–4	**0.026**	4	3–4	3	3–4	0.94
Daily calories (Kcal), median (IQR)	2,025	1,626–2,355	1,880	1,614–2,273	0.49	2,129	1,822–2,475	1,951	1,680–2,379	0.13	1,820	1,590–2,261	1,912	1,637–2,291	0.26	2,050	1,726–2,451	1,975	1,691–2,384	0.92

*BMI, body mass index; ROD, reproductive organ diseases; PA, physical activity; SD, standard deviation; IQR, interquartile range. The bold values indicates p < 0.05*.

To evaluate the strength and direction of the associations among antioxidants intake levels and poor semen quality, we calculated the adjusted ORs (aOR), including potential confounders in the equation, as reported in Table 3 footnotes. Days of abstinence were included in all models.

**Table 3:** Crude and adjusted odds ratios and 95% confidence intervals for selected antioxidant intake.

**Micronutrients:quartiles**	**Semen volume < 1.5 mL^1^**					
	**OR**	**95% CI**	***P***	**aOR**	**95% CI**	***P***
Vitamin D: ref. 1st quartile (10-87)						
2nd (87–122)	0.55	0.27–1.13		0.45	0.20–1.05	
3rd (122–163)	0.28	0.12–0.66		0.28	0.11–0.74	
4th(163–454)	0.35	0.16–0.76		0.25	0.09–0.66	
Chi-square for trend		9.48	0.0002		8.60	0.003
	**Sperm concentration < 15.0 mil/mL^2^**					
	**OR**	**95% CI**	***P***	**aOR**	**95% CI**	***P***
α-carotene: ref. 1st quartile (0.003–0.4)						
2nd (0.4–0.7)	0.57	0.29–1.11		0.44	0.21–0.91	
3^rd^ (0.7–1.2)	0.54	0.28–1.05		0.48	0.23–0.98	
4^th^ (1.2–6.5)	0.51	0.26–1.00		0.43	0.20–0.91	
Chi-square for trend		3.78	0.052		4.39	0.036
Lycopene: ref. 1st quartile (0.5–4)						
2nd (4-6)	0.87	0.42–1.80		0.78	0.36–1.71	
3rd (6-8)	1.37	0.68–2.75		1.54	0.69–3.46	
4th (8-15)	2.22	1.13–4.35		2.46	1.01–5.98	
Chi-square for trend		7.10	0.008		5.53	0.019
	**Sperm motility < 32%^3^**					
	**OR**	**95% CI**	***P***	**aOR**	**95% CI**	***P***
β-cryptoxanthins: ref. 1st quartile (0.00007–0.08)						
2nd (0.08–0.2)	0.67	0.32–1.42		0.71	0.32–1.58	
3rd (0.2–0.3)	0.43	0.19–0.95		0.49	0.16–0.93	
4th (0.3–1)	1.23	0.61–2.48		1.43	0.64–3.20	
Chi-square for trend		0.09	0.76		0.17	0.68
Lutein: ref. 1st quartile (0.05–0.26)						
2nd (0.2–0.3)	0.58	0.28–1.17		0.50	0.23–1.07	
3rd (0.3–0.4)	0.29	0.13–0.65		0.24	0.09–0.62	
4th (0.4–0.9)	0.54	0.26–1.09		0.50	0.19–1.29	
Chi-square for trend		4.54	0.033		3.37	0.06
	**Total sperm count < 39.0 mil^4^**					
	**OR**	**95% CI**	***P***	**aOR**	**95% CI**	***P***
α-carotene: ref. 1st quartile (0.003–0.4)						
2nd (0.4–0.7)	0.60	0.31–1.16		0.53	0.26–1.08	
3rd (0.7–1.2)	0.44	0.22–0.85		0.40	0.20–0.82	
4th (1.2–6.5)	0.48	0.25–0.94		0.46	0.22–0.95	
Chi-square for trend		5.62	0.017		5.41	0.020
β-carotene: ref. 1st quartile (0.5–3)						
2nd (3-4)	0.61	0.31–1.17		0.50	0.25–1.03	
3rd (4-5)	0.66	0.34–1.26		0.41	0.25–1.06	
4th (5-16)	0.49	0.25–0.97		0.51	0.19–0.87	
Chi-square for trend		3.64	0.056		4.86	0.027
Lycopene: ref. 1st quartile (0.5–4)						
2nd (4-6)	0.68	0.33–1.40		0.69	0.32–1.50	
3rd (6-8)	1.22	0.62–2.41		1.71	0.78–3.74	
4th (8-15)	1.97	1.02–3.80		3.11	1.29–7.50	
Chi-square for trend		6.13	0.013		8.62	0.003

*OR, Odds ratio; aOR, adjusted odds ratio; CI, confidence interval.*

*Adjusted for: 1–age class, reproductive organ diseases, alcohol intake, current smoking, daily calories intake, days of abstinence; 2–age class, reproductive organ diseases, alcohol intake, current smoking, daily calories intake, days of abstinence; 3–age class, reproductive organ diseases, occupational physical activity, current smoking, daily calories intake, days of abstinence; 4–age class, reproductive organ diseases, current smoking, daily calories intake, days of abstinence. The cut-off point of quartiles of daily intake for the micronutrients are expressed as mg in brackets*.

Table 3 reports the crude and adjusted ORs of semen volume < 1.5 mL, sperm concentration < 15.0 mil/mL, sperm motility < 32%, and total sperm count < 39.0 mil/mL, according to quartiles of selected micronutrients intake. At the multivariate analysis, we found that risk of low semen volume was lower in men with higher level of vitamin D intake (Chi-square for trend = 8.60, *p* = 0.003). Low sperm concentration was inversely associated with α-carotene level of intake, with similar estimates for 2nd, 3rd, and 4th quartile as compared to the 1st (Chi-square for trend = 4.39, *p* = 0.036).

On the contrary, low sperm concentration was positively associated with lycopene intake (Chi-square for trend = 5.53, *p* = 0.019).

As regards to low sperm motility, lutein and β-cryptoxanthins intakes were not significantly associated in the adjusted analysis. We repeated the analysis considering the quartiles of selected micronutrients intake for kg of body weight, and the results did not change.

## Discussion

In our study, higher α-carotene intake was associated with lower frequency, and lycopene levels with higher frequency of low sperm concentration and total count. Total sperm count was also positively associated with higher β-carotene intake. Vitamin D was positively related to semen volume. These findings were consistent, albeit not always significant, in men with and without history of reproductive organ diseases.

When reading these results, it is important to remember that dietary carotenoids are derived almost completely from fruits and vegetables and vitamin D from milk and dairy products. It is plausible that other components in these foods are responsible for the positive associations with sperm concentration or semen volume, or that the relationships found between micronutrient intake and semen quality may be confounded by other healthy or unhealthy behaviors.

Antioxidants' use has been studied as a possible treatment to reverse the negative impact of high Reactive Oxygen Species (ROS) concentrations on semen quality (22). Many observational studies demonstrated some possible benefits of several types of antioxidants on sperm quality (23–25). But the conclusions are very limited due to the nature of the studies. Preferably, a Cochrane meta-analysis of 48 randomized controlled trials (RCTs) that compared single and combined antioxidants with placebo, in a population of 4,179 sub-fertile men, concluded that there was low quality evidence of a positive effect of antioxidants supplementation on sperm parameters (11).

Regarding the implantation and live birth, a randomized clinical trial of antioxidants supplements, used during infertility treatments, found associations between higher α-carotene intake and lower probability of implantation and live birth, and between higher β-carotene and lower probability of implantation. On the contrary, a positive association was observed between vitamin C and β-carotene intake and fertilization rate (26).

Contrary to our findings, Rahimlou et al. in a cross-sectional study on infertile men with oligospermia have not demonstrated any significant association between lycopene, vitamin E, and α carotene with sperm parameters (27). Furthermore, in a recent placebo-controlled trial, one trial group of infertile men, has been supplemented with lycopene for 12 weeks. Significant improvements in all semen quality parameters and in total antioxidant capacity levels have been found (28). These inconsistent results may be due to differences in study populations (healthy vs. sub-fertile men, smokers vs. non-smokers), sample sizes, and micronutrient intake levels. Also, it should be taken into consideration that the supplementation period is very short if compared with the life of people. A cohort study may be desirable to study the long-term effects of such micronutrients' supplementation on different outcomes.

Dietary patterns may intercept healthy or unhealthy dietary attitudes. Foods that contain compounds important for male fertility belongs to a healthy dietary pattern and are rich in antioxidants, fibres and omega-3 fatty acids, such as fruits, vegetables, legumes, seeds, whole-grain products, nuts, and fish. It is worth mentioning that nutrition should be always considered as a complex system in which all nutrients and foods interact, rather than a unique element that acts positively or negatively on its own (29).

Potential limitations of this study should be considered. As regards the sample size, this cohort study was planned to demonstrate the effect of maternal and paternal diet on ART outcomes and an appropriate sample size calculation was performed (30). The present paper reports a cross-sectional analysis of baseline data from the male partner and no other sample size calculation has been implemented, as regards the present sample.

We assessed dietary, supplement intake and information on lifestyle habits on self-report FFQ. The FFQ is shown in Supplementary Material. Although the FFQ ascertains eating habits over the last year, participants generally “telescope” their report so that their dietary report may reflect recent patterns of intake (31). So, some underestimates due to reporting bias, may have occurred.

Moreover, our results should be referred only to patients of infertile couples and sperm motility was not measured in a subset of patients. Eleven percent of men had sperm concentration lower than 1 million/ml, therefore, the couple was candidate to ICSI; and, according to our center practices, no further analyses were executed, for this reason we did not report a motility value, but only the presence/absence of motility. We did not collect information on vitamin and antioxidant supplementation. The reported frequency of use of antioxidants in a survey conducted during the same calendar period of the present study, on about 800 Italian male internet users, was about 15% (32). This proportion, as suggested by the authors, could be overestimated due to low response rate and potential biases. Furthermore, in Italy, general practitioners do not usually prescribe specific supplements, because male fertility issues are treated by urologists or andrologists. The Italian Society of Andrology and Sexual Medicine (SIAMS) recommended not to prescribe antioxidants for ameliorating sperm parameters and pregnancy rate in absence of a specific diagnosis in all subjects with sperm abnormalities, thus during the study period, supplements were not prescribed as clinical routine in our center (33). Similarly, the interest on the lay press on the role of supplements for improving male fertility is increased more recently, thus it is conceivable that only a very limited proportion of men considered in this study were using supplements. An important limitation regards the possibility of unmeasured confounding. The observed differences might be just due to residual confounding as just four variables were used to adjust the dietary intakes. According to the paper by Smith et al. (34) behavioral, socioeconomic, and physiological factors are strongly interrelated, with 45% of all possible pairwise associations between 96 non-genetic characteristics (*n* = 4,560 correlations being significant at the *p* < 0.01 level). Four confounders are a little number compared with the extensive variations between the lifestyles of people.

Enrolled subjects self-reported information on diet. The questionnaire was considered a reliable tool to measure dietary intake: correlation coefficients were >0.65 for most frequently eaten food, and between 0.50 and 0.65 for others (31, 35). However, the exact amount of each nutrient is difficult to quantify. Although patients reported the frequency of consumption for several foods, the exact micronutrient content in a portion depends on how it is prepared and the size of the portion. As these factors are likely to lower the intake estimates, a systematic bias is likely. According to the paper by Chiu et al. (36), within-patient variability over time was substantial, and a single semen sample may not suffice to correctly classify men as normal according to WHO reference limits.

Some strengths of our study deserve to be commented. Men were interviewed in the same Institution by the same personnel, and participation was practically complete. Moreover, we also accounted for several potential biases, previously reported as associated with semen quality, such as age class, alcohol intake, days of abstinence, and smoking. In summary, although nutrients intake did not eliminate associations between age and semen quality, our results suggest that higher α- and β-carotene and Vitamin D consumption are associated with better sperm parameters and that higher intake of lycopene is associated with a higher frequency of low concentration and total count in our population of sub-fertile men, over a wide age range.

## Conclusion

In our group of male partners of sub-fertile couples undergoing ART cycles, we concluded that a higher intake of α-carotene is positively, and lycopene level is negatively associated with sperm concentration and total count. Besides, higher intake of vitamin D in men is associated with better sperm parameters. Due to the design of the study, we cannot determine a causal relation between selected micronutrient intake and better semen quality, because this may reveal generally healthier lifestyle habits, despite our careful adjustment for alcohol, smoking, and other possibly related covariates.

Further studies, especially well-designed RCTs on the dose-response relations between antioxidants and semen quality parameters, are necessary to confirm the associations found in the present study.

## Data Availability Statement

The raw data supporting the conclusions of this article will be made available by the authors, without undue reservation.

## Ethics Statement

The studies involving human participants were reviewed and approved by the Institutional Review Board (or Ethics Committee) of Fondazione IRCCS Ca' Granda, Ospedale Maggiore, Policlinico, Milan, Italy (Comitato Etico Milano Area B, reference number 2616, Dec. 9, 2014). The patients/participants provided their written informed consent to participate in this study.

## Author Contributions

FP and IL: conceptualization. VD, CA, SN, and SF: data curation. SC, FB, and ER: formal analysis. SN and SF: investigation. FP: methodology. VD and SF: validation. VD and ER: writing – original draft. FP, CA, SN, and GE: writing – review and editing. All authors contributed to the article and approved the submitted version.

## Conflict of Interest

The authors declare that the research was conducted in the absence of any commercial or financial relationships that could be construed as a potential conflict of interest.

## Publisher's Note

All claims expressed in this article are solely those of the authors and do not necessarily represent those of their affiliated organizations, or those of the publisher, the editors and the reviewers. Any product that may be evaluated in this article, or claim that may be made by its manufacturer, is not guaranteed or endorsed by the publisher.
