# Supplementary material for: Antioxidant Vitamins and Carotenoids Intake and the Association With Poor Semen Quality: A Cross-Sectional Analysis of Men Referring to an Italian Fertility Clinic
Source: Front Nutr. 2021 Oct 4;8:737077. doi: 10.3389/fnut.2021.737077 (PMC8520935; doi:10.3389/fnut.2021.737077)
Supplement: Supplementary file 1 [file Table_1.DOCX]

FINAL VERSION

FOOD FREQUENCY QUESTIONNAIRE

CARD 1

DIET **(VARS cond1-cond17; pasti1-pasti5)**

**Study #**	/__/__/	**1-2**
**Patient's #**	/__/__/__/__/__/	**3-7**

THE FOLLOWING QUESTIONS REFER TO YOUR DIETARY HABITS DURING THE YEAR PRIOR TO THE ILLNESS THAT BROUGHT YOU TO THE HOSPITAL

What type of fat do you use to:

1=None	2=Olive oil	3=Sunflower, corn, soy oil
4=Peanuts oil	5= Vegetable oil	6=Butter
7=Margarine	8=Cream, besciamelle, lard, shortening	9=Do not know

	1st type of fat:	2nd type of fat : (if used in equal amount)	
To dress raw vegetables	/__/	/__/	11-12
To cook or season cooked vegetables	/__/	/__/	13-14
To cook meat	/__/	/__/	15-16
To fry	/__/	/__/	17-18
To season pasta or rice (used also for the sauce)	/__/	/__/	19-20

-How would you define your consumption of fat for seasoning?

Low=1; Medium=2; High=3; /__/ **21**

Do you tend to discard the fat in meat?

Yes=1; No=2; No consumption=3; /__/ **22**

Do you tend to discard the fat in prosciutto?

Yes=1; No=2; No consumption=3; /__/ **23**

Do you tend to discard the skin in chicken meat?

Yes=1; No=2; No consumption=3; /__/ **24**

Do you tend to leave the food condiments in your plate?

Yes=1; No=2; No consumption=3; /__/ **25**

How would you define your consumption of garlic?

Scarce =1; Medium=2; High=3; /__/ 26

When eating out, do you add salt to your food?

No=1; Sometimes=2; Always=3; /__/ 27

How many meals do you eat every day? /__/ 28

How many snacks? (including continental breakfast and sandwiches, excluding tea and coffee) /__/ 29

How many meals do you eat at:

Home	/__/__/	30-31
Restaurant	/__/__/	32-33
Canteen	/__/__/	34-35

NOW I WILL ASK YOU ABOUT YOUR DIETARY HABITS DURING THE YEAR PRIOR TO THE ILLNESS THAT HAS BROUGHT YOU TO THE HOSPITAL. YOU SHOULD DESCRIBE THE FREQUENCY OF CONSUMPTION AND THE SERVING SIZE OF SPECIFIED FOOD ITEMS

The weights of the servings are referred to raw food, net weight, except for prepackaged foods (i.e., bread sticks) or when otherwise specified.

Note: Small, is a serving one third smaller than the medium one

Big, is a serving one third bigger than the medium one

Frequency of consumption/week never or <1/month =00; 1-3/month =98; Do not know =99;

Round off : if the person being interviewed gives a frequency of weekly consumption that is not a whole number (for instance 3-4 equals 3.5) it is suggested to invite the subject to choose the number most appropriate. If this is not possible, then round down (i.e., 3.5=3).

(VARS ali1-ali78; porz17-24; porz28-porz43; porz45-51; porz53-57; porz73-74; mesi 52-57; mesi 65-69; mesi78; codop1- 8; open1-8; porop2-5 porop8; rias1-7; porias1-6; reg1-2; codreg1-2; poreg1-2)

Type of food	Frequency		
	Serving	Number/week	
Milk and hot beverages			
Whole milk	1cup, 225ml.	/__ali1__/	36-37
Partially skimmed milk	1cup, 225ml.	/__/__/	38-39
Skimmed milk	1 cup, 225ml.	/__/__/	40-41
Yoghourt	1jar, 125gr.	/__/__/	42-43
Cappuccino (bar)	1 cup, 125ml.	/__/__/	44-45
Coffee	1small cup, 50ml.	/__/__/	46-47
Decaffeinated coffee	1 small cup, 50ml.	/__/__/	48-49
Tea	1 cup, 125ml.	/__/__/	50-51
Sugar	1 teaspoon, 3gr.	/__/__/	52-53
Saccarine	1 packet, tablet, 1.25gr.	/__/__/	54-55
			56-57
Other sweetners	1packet, tablet, 1.25gr.	/__ali11__/	
			58-63
Other hot beverages (>=1 week) specify			
....	codop1	open1	
....	/__/__/__/__/	/__/__/	

CARD 2

DIET

**Study #**	/__/__/	**1-2**
**Patient's #**	/__/__/__/__/__/	**3-7**

**Type of food**	**Serving**	**Quantity** S	**Quantity** M	**Quantity** L	**Frequency** Number/week	
**First courses and cereals**						
**Bread**	**1 slice, 1 roll 50gr.**				/__/__/	**11-12**
**Whole wheat bread**	**1 slice, 1 roll 50gr.**				/__/__/	**13-14**
**Crackers (1packet), bread sticks (5), melba toast (3)**	**30gr.**				/__/__/	**15-16**
**Maize**	**1 slice 100gr.**				/__/__/	**17-18**
**Pizza (cooked) (1 medium size)**	**200gr.**				/__/__/	**19-20**
**Risotto**	**80gr.**		porz17		/__/__/	**21-23**
		/1/	/2/	/3/		
**Pasta/rice seasoned with butter or oil**	**80gr.**	/1/	/2/	/3/	/__/__/	**24-26**
**Pasta/rice with tomato sauce**	**80gr.**	/1/	/2/	/3/	/__/__/	**27-29**
**Pasta/rice with ragù sauce**	**80gr.**	/1/	/2/	/3/	/__/__/	**30-32**
**Pasta/rice with pesto sauce**	**80gr.**	/1/	/2/	/3/	/__/__/	**33-35**
**Lasagne/ cannelloni/ tortellini, with meat filling (cooked)**	**250gr.**	/1/	/2/	/3/	/__/__/	**36-38**
**Light soup with noodles (cooked)**	**250gr.**	/1/	/2/	/3/	/__/__/	**39-41**
**Vegetable soup with noodles (cooked)**	**250gr.**	/1/	/2/	/3/	/__/__/	**42-44**
**Other first courses (>1/week) specify**	/__/__/__/__/__/		porop2		/__/__/	**45-51**
		/1/	/2/	/3/		
....						
**Grated cheese (parmesan type)**	**1 teaspoon**				/__/__/	**52-53**
**In summary**			porias1		rias1	
**pasta/rice with sauce**		/1/	/2/	/3/	/__/__/	**54-56**

**In summary** pasta/rice in soup, with or without vegetables		/1/	/2/	/3/	/__/__/	**57-59**

CARD 3

DIET

Study # /__/__/ **1-2**

Patient's # /__/__/__/__/__/ **3-7**

Type of food	Serving	S	M	L	Number/week	
		Quantity			Frequency	
**Second courses**						
Boiled/ poached or raw eggs	1				/__/__/	11-12
Fried eggs/ omelettes	1				/__/__/	13-14
Chicken/turkey, broiled or boiled	200gr.	/1/	/2/	/3/	/__/__/	15-17
Chicken/turkey, rabbit roasted, fried or stewed	200gr.	/1/	/2/	/3/	/__/__/	18-20
Steak/roast-beef/lean ground beef, veal or horse meat	120gr.	/1/	/2/	/3/	/__/__/	21-23
Boiled beef	150gr.	/1/	/2/	/3/	/__/__/	24-26
Beef or veal stew/meat balls, etc.	150gr.	/1/	/2/	/3/	/__/__/	27-29
Wiener Schinitzel	120gr.	/1/	/2/	/3/	/__/__/	30-32
Pork chop/paillard or pork roast	150gr.	/1/	/2/	/3/	/__/__/	33-35
Liver	150gr.	/1/	/2/	/3/	/__/__/	36-38
Prosciutto (6 slices)/lean processed meat (5)	50gr.	/1/	/2/	/3/	/__/__/	39-41
Ham (3 slices)	50gr.	/1/	/2/	/3/	/__/__/	42-44
Salami (5 slices)/bologna (2 slices)/sausages (1/2), bacon (6 slices), hot dog (1)	50gr.	/1/	/2/	/3/	/__/__/	45-47

Type of food	Serving	S	M	L	Number/week	
		Quantity			Frequency	
Fish/molluscs, boiled or broiled	150gr.	/1/	/2/	/3/	/__/__/	48-50
Fish/molluscs, fried	150gr.	/1/	/2/	/3/	/__/__/	51-53
Tuna (1 small can) /sardines (4 fillets) packed in oil	80gr.	/1/	/2/	/3/	/__/__/	54-56
Ricotta/mozzarella cheese, as second course	100gr.	/1/	/2/	/3/	/__/__/	57-59
Other cheeses as second course	80gr.	/1/	/2/	/3/	/__/__/	60-62
Any type of cheese in addition or as snack	25gr.				/__/__/	63-64
Others, (>=1/week) specify	/__/__/__/__/	/1/	/2/	/3/	/__/__/	65-71
**In summary**						
Red/white meat (excluding processed meat)		/1/	/2/	/3/	/__/__/	72-74
**In summary**						
Fish		/1/	/2/	/3/	/__/__/	75-77

CARD 4

DIET

Study #	/__/__/	1-2
Patient's #	/__/__/__/__/__/	3-7

Type of food	Quantity				Frequency	
	Serving	S	M	L	Number/week	
**Side dishes**						
Peas/beans/chick peas/ lentils (40gr., dry)	100gr.	/1/	/2/	/3/	/__/__/	11-13
Green and red salad	50gr.	/1/	/2/	/3/	/__/__/	14-16
Raw carrots (1medium)	75gr.	/1/	/2/	/3/	/__/__/	17-19
Cooked carrots	130gr.	/1/	/2/	/3/	/__/__/	20-22
Onions (1 medium)	80gr.	/1/	/2/	/3/	/__/__/	23-25
Boiled potatoes (1 medium)	125gr.	/1/	/2/	/3/	/__/__/	26-28
Fried or roasted potatoes	200gr.	/1/	/2/	/3/	/__/__/	29-31
SEASONAL						Months/year
Artichokes (Jan-Apr)	1whole				/__/__/	/__mesi52__/ 32-35
Cruciferae (Nov-Apr)	125gr.	/1/	/2/	/3/	/__/__/	/__/__/ 36-40
Spinach/other greens (Nov-Apr)	200gr.	/1/	/2/	/3/	/__/__/	/__/__/ 41-45
Tomatoes (Apr-Sept), (1 medium)	150gr.	/1/	/2/	/3/	/__/__/	/__/__/ 46-50
Salad with carrots, cucumbers, peppers (Apr-Sept)	100gr.	/1/	/2/	/3/	/__/__/	/__/__/ 51-55
Zucchini/eggplants/peppers, cooked (Apr-Sept)	150gr.	/1/	/2/	/3/	/__/__/	/__/__/ 56-60

Other side dishes (>=20 times/year) specify:	/__/__/__/__/	/1/	/2/	/3/	/__/__/	/__/__/ 61-69
Other side dishes (>=20 times/year) specify:..	/__/__/__/__/	/1/	/2/	/3/	/__/__/	/__/__/ 70-78
**In summary** Raw vegetables		/1/	/2/	/3/	/__/__/	79-81
**In summary** Cooked vegetables (excluding potatoes)		/1/	/2/	/3/	/__/__/	82-84

	Serving	S	M	L	Number/week	
1° Course regional recipe specify:..................................	/__/__/__/__/	/1/	/2/	/3/	/__/__/	85-91
2° Course regional recipe specify:..................................	/__/__/__/__/	/1/	/2/	/3/	/__/__/	92-98

CARD 5
DIET

-

Study # /__/__/ **1-2**
Patient # /__/__/__/__/__/ **3-7**

Type of food	Frequency		
	Serving	Number/week	
**Fruits**			
**Apples and pears**	**1 med. size**	/__/__/	**11-12**
**Bananas**	**1 med. size**	/__/__/	**13-14**
**Kiwi**	**1 med. size**	/__/__/	**15-16**
**Cooked fruits**	**150gr.**	/__/__/	**17-18**
**Fruit juices (unsweetened)**	**150ml.**	/__/__/	**12-20**
**Fruit juices, sweetened (small bottle)**	**125gr.**	/__/__/	**21-22**
**Honey or jam**	**1teaspoon 3gr.**	/__/__/	**23-24**

Type of food	Frequency			
	Serving	Number/week		
**Fruit**				
**SEASONAL**			**Months**	
**Citrus fruits (not spec: 6 months, Nov-Apr.)**	**150gr.**	/__/__/	/__/__/	**25-28**
**Peaches, apricots and prunes (not spec: 4 months, Jun-Aug)**	**100gr.**	/__/__/	/__/__/	**29-30**
**Melon (not spec: 3 months, Jun-Aug)**	**2 slices 75gr.**	/__/__/	/__/__/	**33-36**
**Grapes (not spec: 3 months, Aug-Oct)**	**230 gr.**	/__/__/	/__/__/	**37-40**
**Strawberries and cherries (not spec: 2 months, May-Jun)**	**1small bowl 150gr.**	/__/__/	/__/__/	**41-44**
**Other fruits (at least 20 times a year) specify:..**	/__/__/__/__/__/	/__/__/	/__/__/	**45-52**
**Other fruits (at least 20 times a year) specify:..**	/__/__/__/__/__/	/__/__/	/__/__/	**52-60**
**In summary**				
**Fresh fruit**		/__/__/		**61-62**

Type of food	Serving	Quantity S	M	L	Frequency Number/week	
Biscuits	7				/__/__/	63-64
	50gr.					
Croissants and doughnuts	50 gr.				/__/__/	65-66
Pastry, doughnuts with cream or	50gr.				/__/__/	67-68
custard	1 slice	/1/	/2/	/3/	/__/__/	69-71
Pound cakes, plain cakes, christmas and	100gr.					
easter cakes	100gr.	/1/	/2/	/3/	/__/__/	72-74
Fruit or jam pies (1 slice), fruit tarts (1)	1				/__/__/	75-76
Chocolate candies	1 glass				/__/__/	77-78
Soft drinks	150ml.					
	1				/__/__/	79-80
Candies	2 scoops				/__/__/	Months /__/__/
Icecream	1cone, 100gr.					81-84
Other cakes or soft drinks (=1/week) specify:..	/__/__/__/__/__/	/1/	/2/	/3/	/__/__/	85-91

CARD 6

V – DIET (VARS alc1-27)

Study # /__/__/ 1-2

Patient # /__/__/__/__/__/ 3-7

ALCOHOL CONSUMPTION

- Do you drink or used to drink alcoholic beverages?

/1/ No /2/ Yes /3/ Only in the past alc1 /__/ 11

When?

/1/ With meals /2/ Between meals /3/ Both alc2 /__/ 12

Type of drink	No. days/week	No. units/week	Age at beginning of regular consumption	If ex drinker, give age at quitting	
Wine (125ml.)	alc3 /__/	alc4 /__/__/__/	alc5 /__/__/	alc6 /__/__/	13-20
Beer (330ml.)	alc7 /__/	alc8 /__/__/__/	alc9 /__/__/	alc10 /__/__/	21-28
After dinner drinks (30ml.)	alc11 /__/	alc12 /__/__/__/	alc13 /__/__/	alc14 /__/__/	29-36
Grappa (30ml.)	alc15 /__/	alc16 /__/__/__/	alc17 /__/__/	alc18 /__/__/	37-44
Whisky, cognac and brandy (30ml.)	alc19 /__/	alc20 /__/__/__/	alc21 /__/__/	alc22 /__/__/	45-52
Others, (=1/week) specify:					
/__/__/__/__/	/__/	/__/__/__/	/__/__/	/__/__/	53-64

Note: Consumption 1-3/month = 998
